# Supplementary material for: Orthodenticle homeobox 2 is transported to lysosomes by nuclear budding vesicles
Source: Nat Commun. 2023 Feb 27;14:1111. doi: 10.1038/s41467-023-36697-5 (PMC9971051; doi:10.1038/s41467-023-36697-5)
Supplement: Supplementary file 1 — Supplementary Information [file 41467_2023_36697_MOESM1_ESM.pdf]

Supplementary Information

Orthodenticle homeobox 2 is transported to lysosomes by nuclear budding vesicles

Jun Woo Park *et al*.

- Supplementary Figures 1 – 8
- Supplementary Tables 1 – 2

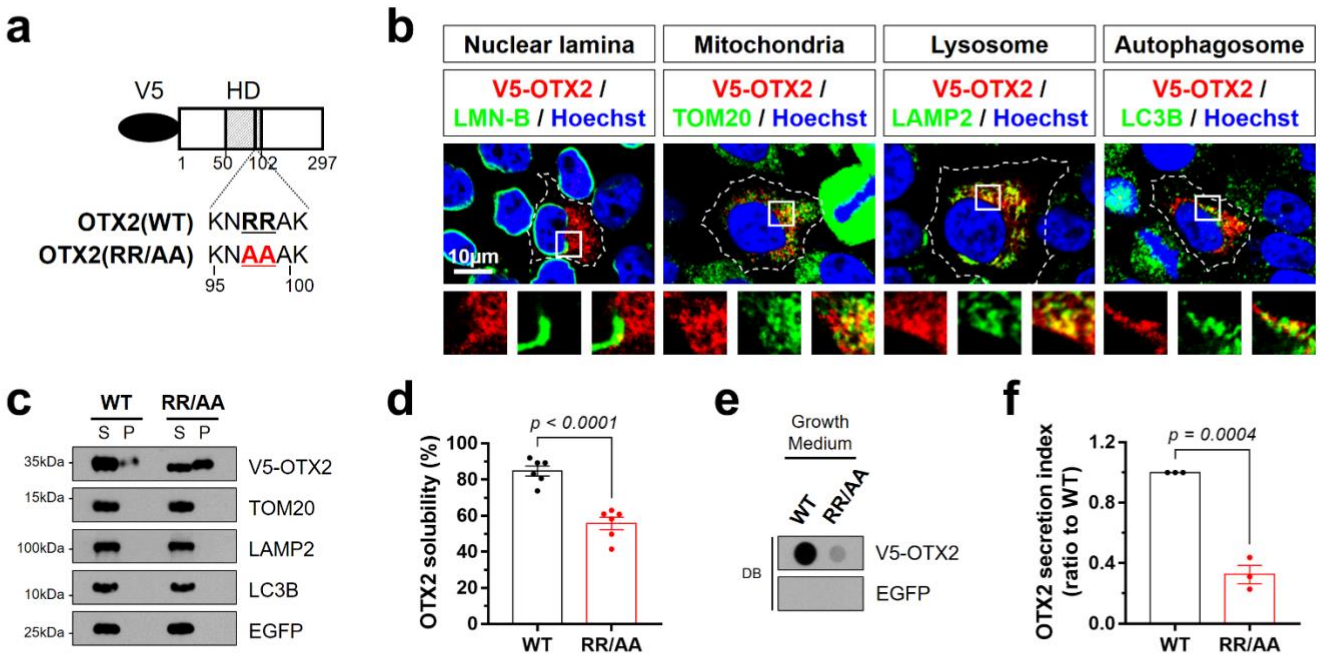

Supplementary Fig. 1. Cytoplasmic OTX2 puncta originate from the nucleus. **a**, Diagram exhibiting V5-tagged human OTX2 (top) and its NLS including R97 and R98, which are mutated to A97 and A98 in OTX2(RR/AA) mutant. **b**, HeLa cells overexpressing V5-OTX2(RR/AA) were stained with antibodies against V5 and the organelle-specific markers. Nuclei of the cells were also visualized by staining with Hoechst. Similar observations were made in 3 independent experiments, and representative images are shown. **c**, HeLa cells overexpressing V5-OTX2(WT) and V5-OTX2(RR/AA) were lysed in RIPA buffer. Then, soluble supernatant (S) and insoluble precipitate (P) fractions were separated by centrifugation. V5-OTX2 and co-expressed EGFP in those fractions were detected by WB. Markers for mitochondria (TOM20), lysosome (LAMP2), and autophagosome (LC3B) were also examined by WB. **d**, OTX2 solubility was determined by dividing the band intensity of V5-OTX2 in S fraction by that of total (S+P) and shown in the graph. The values are the means and error bars denote SEM obtained from 6 independent experiments. **e**, V5-tagged OTX2 proteins and EGFP in the growth media of the cells were determined by DB with anti-V5 and anti-GFP antibodies. **f**, The graph shows mean relative secretion index values. Error bars denote SEM obtained from 3 independent experiments. Statistical analysis (in **d** and **f**) was performed by Student's t-test (two-tailed), and source data are provided as a Source Data file.

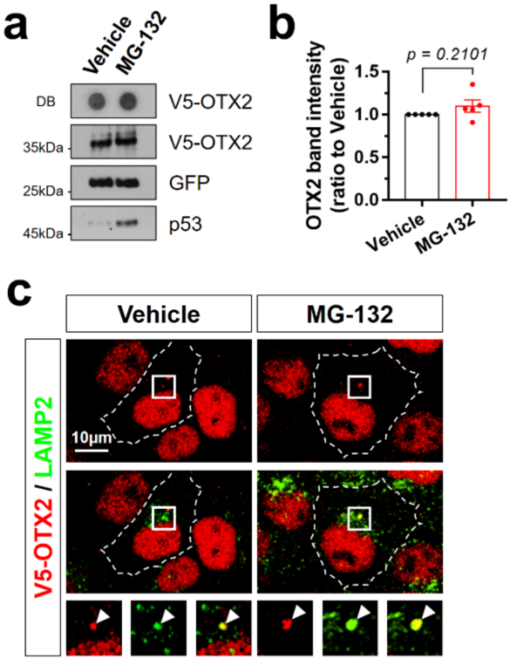

Supplementary Fig. 2. OTX2 is insensitive to proteasomal degradation. a, OTX2-HeLa cells were treated with either vehicle (DMSO) or MG-132 (10μM) for 4hr. Intracellular V5-OTX2 were detected WB with anti-V5 antibody. Relative levels of OTX2 expressed in the samples were examined by comparing the levels of EGFP, which is expressed together with OTX2 from a same mRNA. The levels of p53 in these cells were also examined by WB with anti-p53 antibody to confirm the inhibition of proteasomal protein degradation by MG-132. **b**, The graph shows mean values of OTX2 WB band intensity, which were divided by that of EGFP in the same sample. Error bars denote SEM obtained from 5 independent experiments. Statistical analysis was performed by two-tailed Student's t-test, and source data are provided as a Source Data file. **c**, Lysosomal localization of V5-OTX2 in OTX2-HeLa cells, which were treated with vehicle or MG-132, was investigated by immunostaining with anti-V5 and anti-LAMP2 antibodies. Arrowheads indicate the cytoplasmic V5-OTX2 puncta colocalizing with LAMP2. Similar observations were made in 5 independent experiments, and representative images are shown.

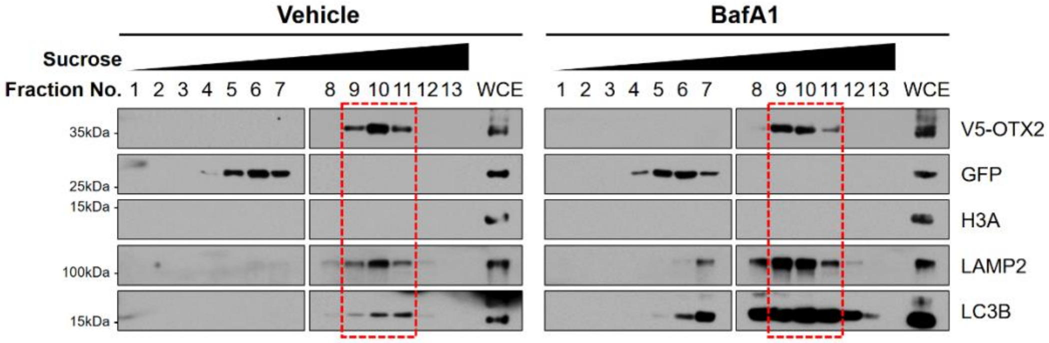

Supplementary Fig. 3. Cytoplasmic OTX2 is located in the lysosomal fraction. OTX2-HeLa cells were treated with or without BafA1 (200nM for 24hr) prior to the separation into cytoplasmic and nuclear fractions. Cytoplasmic fractions (90% of total lysates) of OTX2-HeLa cells were further separated by sucrose density gradient centrifugation. Proteins in 5% of each fraction and 0.2% of WCE were detected by WB. Dotted red box indicates OTX2-positive fractions. Similar results were obtained in 3 independent experiments, and representative blots are shown.

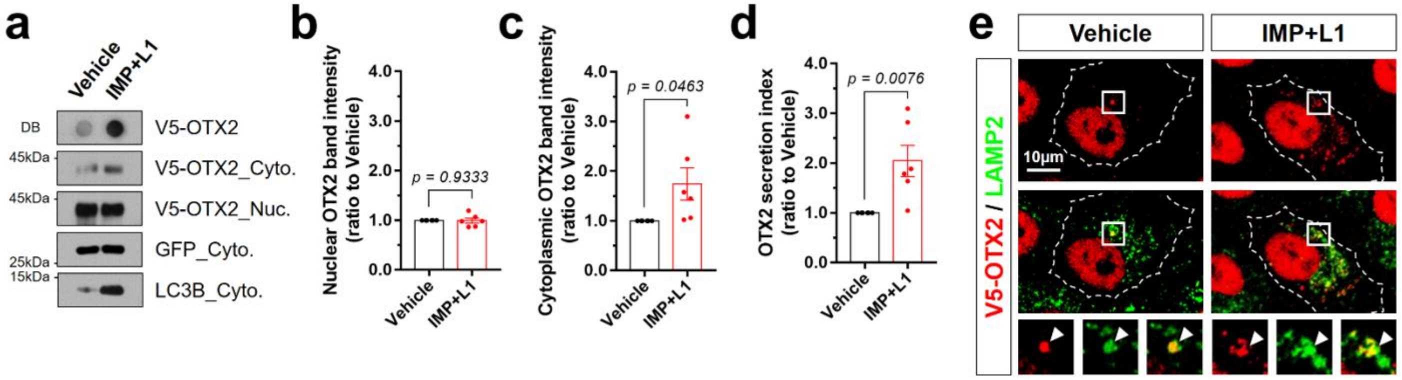

Supplementary Fig. 4. OTX2 is sensitive to lysosomal lipases. a, OTX2-HeLa cells were treated with the cocktail of lysosomal lipase inhibitors, IMP (50μM) and L1 (10μM), or vehicle (0.05% methanol and 7.04mM DMSO in DMEM) for 24h. V5-OTX2 secreted into the growth medium of the cells was detected by dot blotting (DB) with anti-V5 antibody. Cytoplasmic (Cyto.) and nuclear (Nuc.) fractions of OTX2-HeLa cells were separate as described in Methods. Levels of V5-OTX2 in each fraction were then detected by western blotting (WB). Inhibition of lysosomal lipase function by IMP and L1 was confirmed by detecting the accumulation of LC3B examined by WB. **b** and **c**, The graphs show relative intensities of WB bands of nuclear (**b**) and cytoplasmic OTX2 (**c**). **d**, The graph shows relative secretion index of OTX2 determined by DB. The columns represent means and error bars denote SEM. Results were obtained from 6 independent experiments. **e**, OTX2-HeLa cells treated with vehicle or the lysosomal lipase inhibitors were stained with antibodies that recognize V5 and LAMP2. Images in the bottom row are magnified versions of the boxed areas in top row. Arrowheads indicate LAMP2-positive cytoplasmic puncta containing V5-OTX2. Similar observations were made in 3 independent experiments, and representative images are shown. Two-tailed Student's t-test was performed for the statistical analysis (in **b**–**d**), and source data are provided as a Source Data file.

a

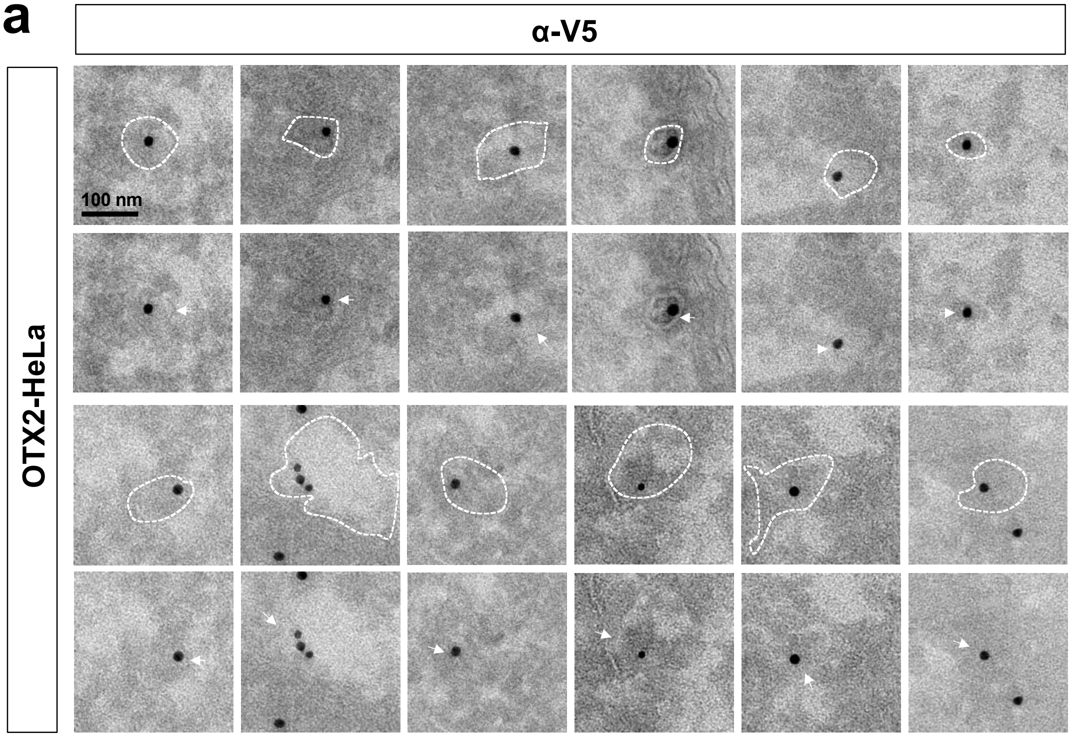

b

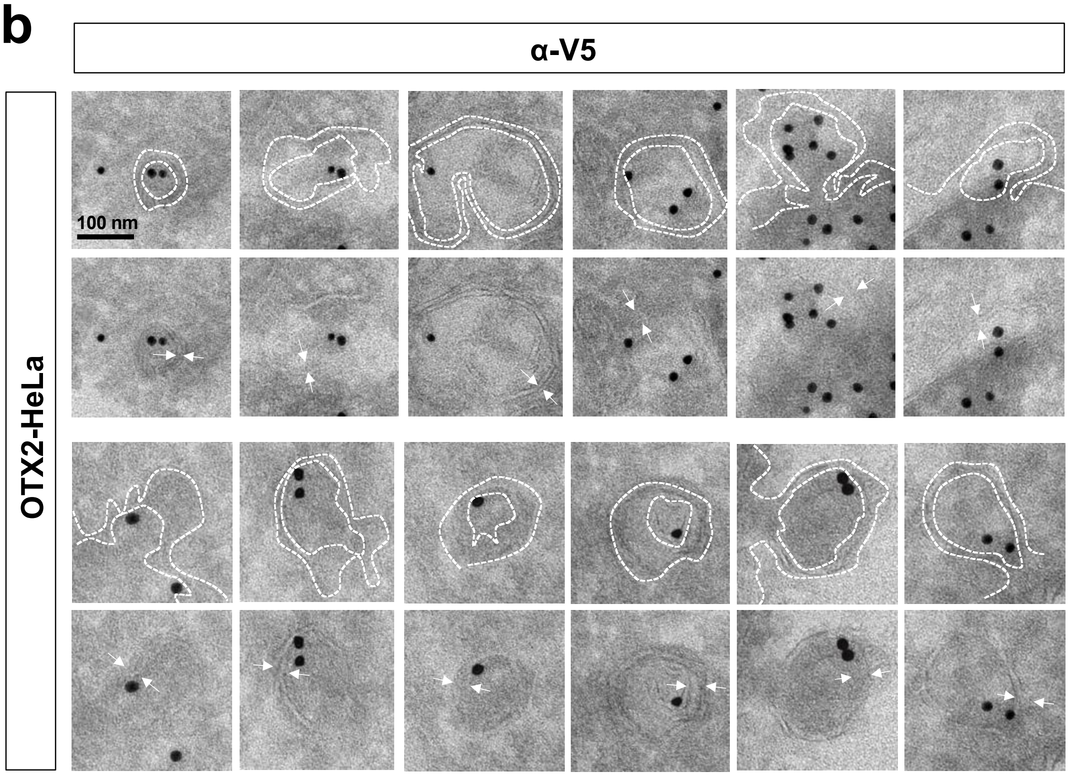

Supplementary Fig. 5. iTEM images of V5-tagged OTX2 in HeLa cells. Cryo-sections (50nm) of OTX2-HeLa cells were stained with gold-labeled mouse anti-V5 antibody (α-V5) to visualize V5-OTX2 on the sections by TEM. Representative iTEM images of single- (a) and double-layered (b) membrane vesicles are provided. White dotted lines in the upper rows indicate vesicular membranes. Arrows in the bottom rows point the inner and outer vesicular membranes. These images are used for the quantifications in Fig. 3c.

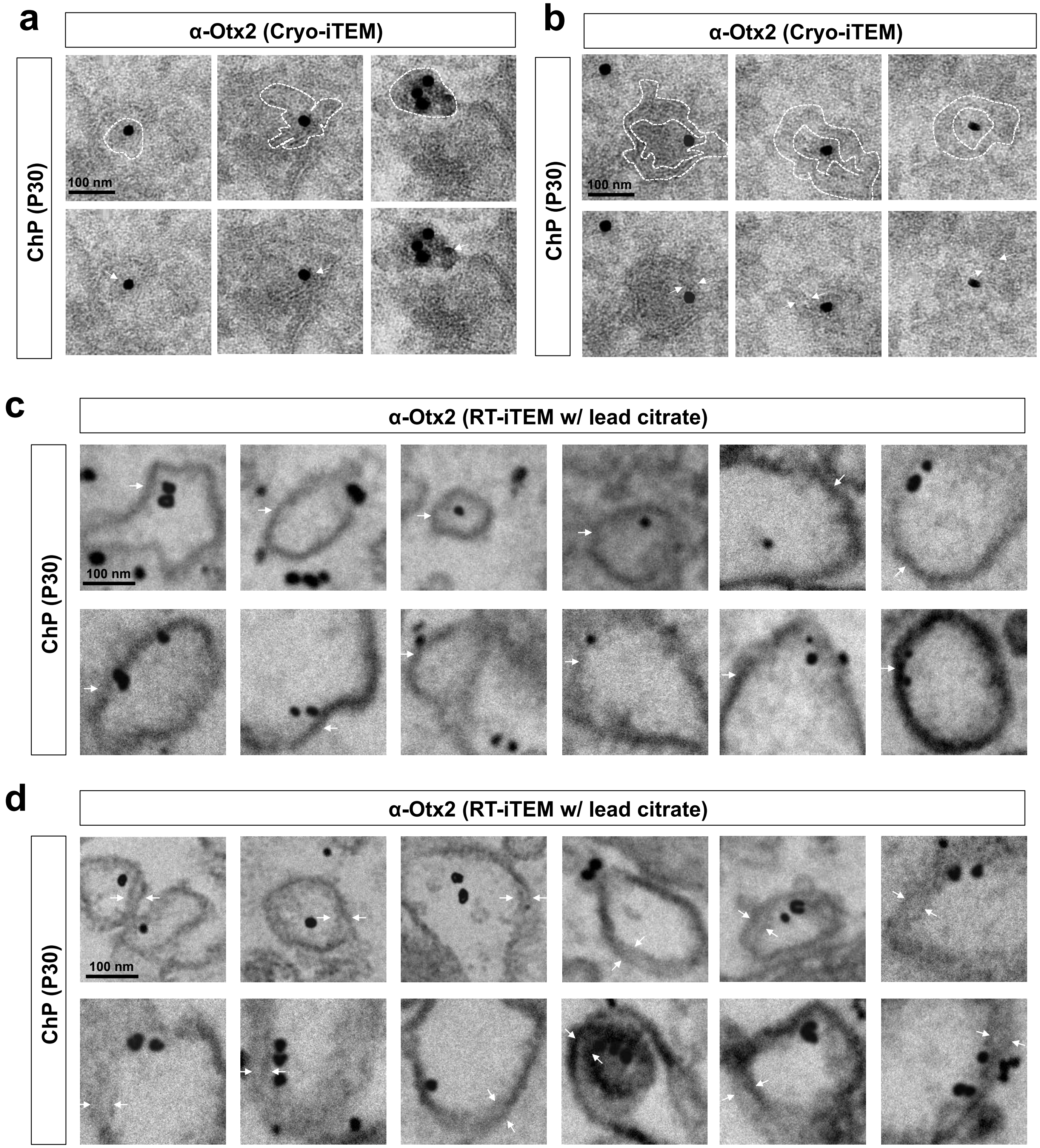
a
α-Otx2 (Cryo-iTEM)
ChP (P30)
100 nm
b
α-Otx2 (Cryo-iTEM)
ChP (P30)
100 nm
c
α-Otx2 (RT-iTEM w/ lead citrate)
ChP (P30)
100 nm
d
α-Otx2 (RT-iTEM w/ lead citrate)
ChP (P30)
100 nm

Supplementary Fig. 6. iTEM images of Otx2 in mouse ChP cells. Cryo-sections (a, b) or RT-sections (c, d) of P30 C57BL/6J mouse ChP were stained with rabbit anti-Otx2 IgG and then gold-labeled anti-rabbit IgG as described in Methods. Representative Otx2 iTEM images of single- (a, c) and double-layered (b, d) membrane vesicles in the ChP cells are provided in this figure. The images in (c) and (d) are obtained from the sections counterstained with lead citrate that visualizes electron-dense areas including vesicular membranes, whereas those in (a) and (b) were not. White dotted lines in the upper rows in (a) and (b) indicate vesicular membranes. Arrows in the bottom rows point the inner and outer vesicular membranes. These images are used for the quantifications in Fig. 3h.

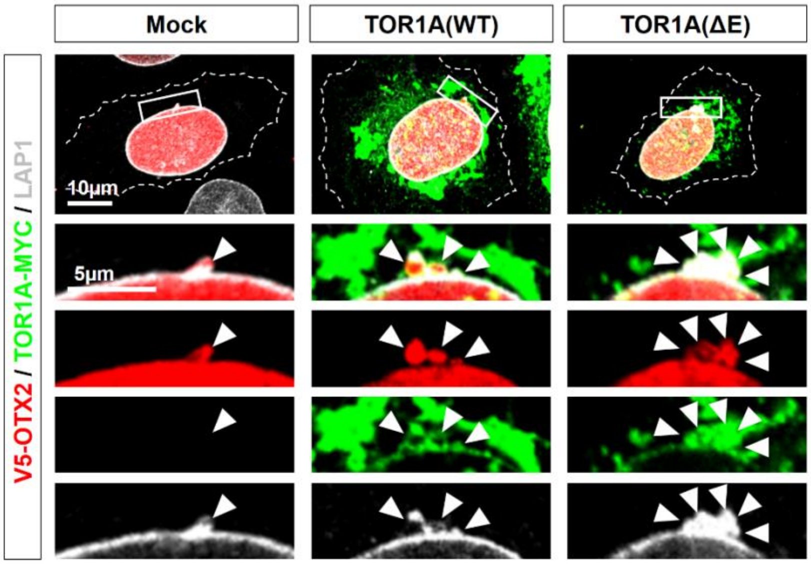

Supplementary Fig. 7. Accumulation of OTX2 in nuclear membrane buds in the cells expressing TOR1AΔE. HeLa cells overexpressing V5-OTX2 and MYC-tagged human TOR1A(WT) or TOR1AΔE were stained with antibodies detecting V5 and MYC. LAP1 in the inner nuclear membrane of the cells was also visualized by immunostaining with anti-LAP1 antibody. Arrowheads point to the nuclear membrane buds containing V5-OTX2. The images in three bottom rows are magnified versions of boxed areas in the top row. Dotted lines are the edges of the cells. Similar observations were made in 3 independent experiments, and representative images are shown.

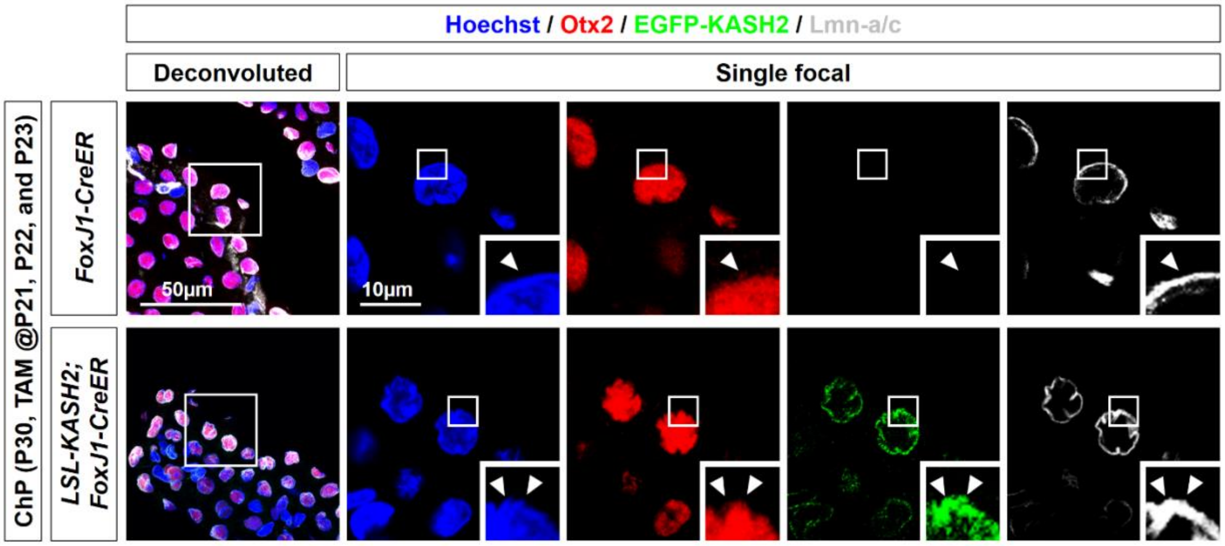

Supplementary Fig. 8. Expression of EGFP-KASH2 in ChP cells of *LSL-KASH2;FoxJ1-CreER* mice. Sections of P30 *FoxJ1-CreER* and *LSL-KASH2;FoxJ1-CreER* littermate mouse brains were stained with anti-Otx2, anti-Lmn-a/c, and anti-GFP antibodies. Nuclei of the cells were stained by Hoechst. Images in right four columns are magnified versions of the boxed areas in the leftmost column. Insets are magnified versions of the boxed areas in the corresponding images. Arrowheads point to the margin of internuclear membrane spaces, which are filled with EGFP-KASH2. Similar observations were made in 3 independent experiments, and representative images are shown.

Supplementary Table 1. List of pharmacological inhibitors used in this study

Chemical	Working concentration	Incubation time	Manufacturer (catalog #)
Bafilomycin A1	200nM	24hours	Sigma-Aldrich (B1793)
Chloroquine diphosphate salt	50μM	24hours	Sigma-Aldrich (C6628)
Dynamin Inhibitor I, Dynasore	50μM	12hours	Sigma-Aldrich (324410)
Dynamin Inhibitor II, MiTMAB	10μM	12hours	Sigma-Aldrich (324411)
Imipramine	50μM	24hours	Sigma-Aldrich (I7379)
Lalistat 1	10μM	24hours	Tocris Bioscience (6098)
(R)-MG132	10μM	4hours	Sigma-Aldrich (M8699)

Supplementary Table 2. List of antibodies used in this study

Antibody	Dilution	Manufacturer (catalog #)
Mouse monoclonal anti α-Tubulin (B-7)	WB (1:2,000)	Santa Cruz (sc-5286)
Mouse monoclonal anti β-Actin (C4)	WB (1:2,000)	Santa Cruz (sc-47778)
Rabbit monoclonal anti Calnexin (C5C9)	ICC (1:100)	Cell Signaling (#2679)
Mouse monoclonal anti-Calretinin (clone 6B8.2)	WB (1:1,000); IHC (1:100)	Sigma-Aldrich (MAB1568)
Mouse monoclonal anti-CD63 (H5C6)	ICC (1:100)	Novus (NBP2-42225)
Rabbit polyclonal anti-Cleaved Caspase-3 (Asp175)	IHC (1:100)	Cell Signaling (#9661)
Mouse monoclonal anti-EEA1	ICC (1:100)	BD Biosciences (610456)
Rabbit monoclonal anti-EGF Receptor (D38B1)	ICC (1:100)	Cell Signaling (#4267)
Rabbit polyclonal anti-Flotillin 1	WB (1:500); ICC (1:100)	Abcam (ab41927)
Mouse monoclonal anti-GFP (B-2)	WB (1:2,000); ICC (1:100)	Santa Cruz (sc-9996)
Rat monoclonal anti-GFP (1A5)	IHC (1:100)	Santa Cruz (sc-101536)
Rabbit monoclonal anti-GM130 (EP892Y)	ICC (1:100)	Abcam (ab52649)
Rabbit polyclonal anti-Histone H3	WB (1:2,000)	Abcam (ab1791)
Mouse monoclonal anti-IGF-I (H-9)	WB (1:1,000)	Santa Cruz (sc-518040)
Rabbit polyclonal anti-IGF-I Receptor β	WB (1:1,000)	Cell Signaling (#3027)
Mouse monoclonal anti-Lamin A/C (E-1)	IHC (1:100)	Santa Cruz (sc-376248)
Rabbit monoclonal anti-Lamin A + Lamin C	WB (1:1,000); ICC (1:100)	Abcam (ab108595)
Mouse monoclonal anti-LAMP-2 (H4B4)	WB (1:1,000)	Santa Cruz (sc-18822)
Goat polyclonal anti-LAMP-2/CD107b	ICC (1:1,000)	R&D (AF6228)
Rabbit polyclonal anti-LAP1B	WB (1:1,000)	Novus (NBP2-47403)
Rabbit polyclonal anti-LC3B	WB (1:1,000); ICC (1:100)	Cell Signaling (#2775)
Mouse monoclonal anti-c-Myc (9E10)	WB (1:1,000); ICC (1:100)	Santa Cruz (sc-40)
Rabbit monoclonal anti-Otx2 (EPR20375)	WB (1:1,000); iTEM (1:100)	Abcam (ab183951)
Goat polyclonal anti-Otx2	IHC (1:100)	R&D (AF1979)
Rabbit polyclonal anti-p53 (FL-393)	WB (1:1,000)	Santa Cruz (sc-6243)
Rabbit polyclonal anti-Parvalbumin	WB (1:1,000); IHC (1:100)	Novus (NB120-11427)
Rabbit polyclonal anti-RFP	WB (1:1,000); ICC (1:100)	Abcam (ab62341)
Rabbit polyclonal anti-SUN1	WB (1:1,000)	Novus (NBP1-87396)
Rabbit polyclonal anti-Syndecan 3	WB (1:100)	Abcam (ab36653)
Rabbit polyclonal anti-Nesprin-2	WB (1:1,000)	Sigma-Aldrich (ABT182)
Rabbit polyclonal anti-Nesprin 2	ICC (1:100)	Novus (NBP1-84190)
Rabbit polyclonal anti-Tom20 (FL-145)	ICC (1:1,000)	Santa Cruz (sc-11415)

Rabbit polyclonal anti-Torsin A	WB (1:1,000);<br>ICC (1:100);<br>IHC (1:100)	Novus (NBP2-95160)
Mouse monoclonal anti-V (OASA04487)	DB (1:1,000);<br>WB (1:1,000);<br>ICC (1:100);<br>iTEM (1:100)	Aviva Systems Biology (OASA04487)
Biotin-conjugated anti-Wisteria floribunda agglutinin	IHC (1:100)	Sigma-Aldrich (L1516)
HRP Rabbit anti-Goat IgG (H+L)	WB (1:10,000)	Jackson ImmunoResearch (31402)
HRP Goat anti-Mouse IgG (H+L)	WB (1:10,000)	Jackson ImmunoResearch (31430)
HRP Goat anti-Rabbit IgG (H+L)	WB (1:10,000)	Jackson ImmunoResearch (31460)
Alexa Fluor® 488 Donkey anti-Chicken IgG (H+L)	ICC (1:100);<br>IHC (1:100)	Jackson ImmunoResearch (703-546-155)
Alexa Fluor® 488 Donkey anti-Goat IgG (H+L)	ICC (1:100);<br>IHC (1:100)	Jackson ImmunoResearch (705-546-147)
Alexa Fluor® 488 Donkey anti-Mouse IgG (H+L)	ICC (1:100);<br>IHC (1:100)	Jackson ImmunoResearch (715-546-150)
Alexa Fluor® 488 Donkey anti-Rat IgG (H+L)	IHC (1:100)	Jackson ImmunoResearch (712-546-150)
Alexa Fluor® 647 Donkey anti-Goat IgG (H+L)	ICC (1:100);<br>IHC (1:100)	Jackson ImmunoResearch (705-606-147)
Alexa Fluor® 647 Donkey anti-Mouse IgG (H+L)	ICC (1:100);<br>IHC (1:100)	Jackson ImmunoResearch (715-606-150)
Alexa Fluor® 647 Donkey anti-Rabbit IgG (H+L)	ICC (1:100);<br>IHC (1:100)	Jackson ImmunoResearch (711-606-152)
Cy™3 Donkey anti-Mouse IgG (H+L)	ICC (1:100);<br>IHC (1:100)	Jackson ImmunoResearch (715-166-150)
Cy™3 Donkey anti-Rabbit IgG (H+L)	ICC (1:100);<br>IHC (1:100)	Jackson ImmunoResearch (711-166-152)
Alexa Fluor® 647 Streptavidin	IHC (1:100)	Jackson ImmunoResearch (016-600-084)
Protein A-Gold 20 nm	iTEM (1:100)	UMC Utrecht (PAG 20 nm)
