# Supplementary material for: Orthodenticle homeobox 2 is transported to lysosomes by nuclear budding vesicles
Source: Nat Commun. 2023 Feb 27;14:1111. doi: 10.1038/s41467-023-36697-5 (PMC9971051; doi:10.1038/s41467-023-36697-5)
Supplement: Supplementary file 2 — Reporting Summary [file 41467_2023_36697_MOESM2_ESM.pdf]

nature portfolio

Corresponding author(s): Jin Woo Kim

Last updated by author(s): Jan 17, 2023

Reporting Summary

Nature Portfolio wishes to improve the reproducibility of the work that we publish. This form provides structure for consistency and transparency in reporting. For further information on Nature Portfolio policies, see our Editorial Policies and the Editorial Policy Checklist.

Statistics

For all statistical analyses, confirm that the following items are present in the figure legend, table legend, main text, or Methods section.

n/a	Confirmed	
☐	☒	The exact sample size (*n*) for each experimental group/condition, given as a discrete number and unit of measurement
☐	☒	A statement on whether measurements were taken from distinct samples or whether the same sample was measured repeatedly
☐	☒	The statistical test(s) used AND whether they are one- or two-sided<br>*Only common tests should be described solely by name; describe more complex techniques in the Methods section.*
☐	☒	A description of all covariates tested
☐	☒	A description of any assumptions or corrections, such as tests of normality and adjustment for multiple comparisons
☐	☒	A full description of the statistical parameters including central tendency (e.g. means) or other basic estimates (e.g. regression coefficient) AND variation (e.g. standard deviation) or associated estimates of uncertainty (e.g. confidence intervals)
☐	☒	For null hypothesis testing, the test statistic (e.g. F, t, r) with confidence intervals, effect sizes, degrees of freedom and P value noted<br>*Give P values as exact values whenever suitable.*
☒	☐	For Bayesian analysis, information on the choice of priors and Markov chain Monte Carlo settings
☒	☐	For hierarchical and complex designs, identification of the appropriate level for tests and full reporting of outcomes
☒	☐	Estimates of effect sizes (e.g. Cohen's *d*, Pearson's *r*), indicating how they were calculated

Our web collection on statistics for biologists contains articles on many of the points above.

Software and code

Policy information about availability of computer code

Data collection	Olympus FV3000 RS Fluoview software; ImageJ software (NIH, v.1.53c); OptoMotry software (CerebralMechanics, v.2.0.0)
Data analysis	Prism 7 software (GraphPad Software, v.7.00)

For manuscripts utilizing custom algorithms or software that are central to the research but not yet described in published literature, software must be made available to editors and reviewers. We strongly encourage code deposition in a community repository (e.g. GitHub). See the Nature Portfolio guidelines for submitting code & software for further information.

Data

Policy information about availability of data

All manuscripts must include a data availability statement. This statement should provide the following information, where applicable:

- Accession codes, unique identifiers, or web links for publicly available datasets
- A description of any restrictions on data availability
- For clinical datasets or third party data, please ensure that the statement adheres to our policy

All data generated or analysed during this study are included in this published article (and its supplementary information files).

Human research participants

Policy information about studies involving human research participants and Sex and Gender in Research.

Reporting on sex and gender	None
Population characteristics	None
Recruitment	None
Ethics oversight	None

Note that full information on the approval of the study protocol must also be provided in the manuscript.

Field-specific reporting

Please select the one below that is the best fit for your research. If you are not sure, read the appropriate sections before making your selection.

☒ Life sciences ☐ Behavioural & social sciences ☐ Ecological, evolutionary & environmental sciences

For a reference copy of the document with all sections, see nature.com/documents/nr-reporting-summary-flat.pdf

Life sciences study design

All studies must disclose on these points even when the disclosure is negative.

Sample size	Sample sizes were determined based on previous study with similar experiments (Lee et al., 2019; https://doi.org/10.1016/j.celrep.2019.06.056). Sample size information is provided in each figure legend. For quantifications from microscopy images, a minimum of n=50 cells were counted across sections although more cells were counted if available in the field of view. All results in this study were obtained from at least 3 independent experiments.
Data exclusions	None
Replication	For each experiment the number of independent sample is described in each figure legend. Independent experiments were performed at least 3 times.
Randomization	For in vitro experiment, samples were treated in random order. For animal experiment, littermate mice were randomly allocated to experimental and control groups, and mice were exposed to the same environmental condition.
Blinding	Sample selection and quantitative analyses of the data were done a blinded fashion, and the samples were matched after quantification.

Reporting for specific materials, systems and methods

We require information from authors about some types of materials, experimental systems and methods used in many studies. Here, indicate whether each material, system or method listed is relevant to your study. If you are not sure if a list item applies to your research, read the appropriate section before selecting a response.

Materials & experimental systems

n/a	Involved in the study	
☐	☒	Antibodies
☐	☒	Eukaryotic cell lines
☒	☐	Palaeontology and archaeology
☐	☒	Animals and other organisms
☒	☐	Clinical data
☒	☐	Dual use research of concern

Methods

n/a	Involved in the study	
☒	☐	ChIP-seq
☒	☐	Flow cytometry
☒	☐	MRI-based neuroimaging

Antibodies

Antibodies used	Detailed information on antibodies used in this study is provided in Supplementary Table 2.
Validation	All antibodies used in this study have been validated by the manufacturers for species and application.

Validation

Validation information is available from the manufacturer's website.

α-Tubulin (B-7), Santa Cruz, Cat#: sc-5286
https://www.scbt.com/p/alpha-tubulin-antibody-b-7
β-Actin (C4), Santa Cruz, Cat#: sc-47778
https://www.scbt.com/p/beta-actin-antibody-c4
anti Calnexin (C5C9), Cell Signaling, Cat#: 2679
https://www.cellsignal.com/products/primary-antibodies/calnexin-c5c9-rabbit-mab/2679
anti-Calretinin (clone 6B8.2), Sigma-Aldrich, Cat#: MAB1568
https://www.sigmaaldrich.com/KR/ko/product/mm/mab1568
anti-CD63 (H5C6), Novus, Cat#: NBP2-42225
https://www.novusbio.com/products/cd63-antibody-h5c6_nbp2-42225
anti-Cleaved Caspase-3 (Asp175), Cell Signaling, Cat#: 9661
https://www.cellsignal.com/products/primary-antibodies/cleaved-caspase-3-asp175-antibody/9661
anti-EEA1, BD Biosciences, Cat#: 610456
https://www.bdbiosciences.com/en-au/products/reagents/microscopy-imaging-reagents/immunofluorescence-reagents/purified-mouse-anti-eea1.610456
anti-EGF Receptor (D38B1), Cell Signaling, Cat#: 4267
https://www.cellsignal.com/products/primary-antibodies/egf-receptor-d38b1-xp-rabbit-mab/4267
anti-Flotillin 1, Abcam, Cat#: ab41927
https://www.abcam.com/flotillin-1-antibody-ab41927.html
anti-GFP (B-2), Santa Cruz, Cat#: sc-9996
https://www.scbt.com/p/gfp-antibody-b-2
anti-GFP (1A5), Santa Cruz, Cat#: sc-101536
https://www.scbt.com/p/gfp-antibody-1a5
anti-GM130 (EP892Y), Abcam, Cat#: ab52649
https://www.abcam.com/gm130-antibody-ep892y-cis-golgi-marker-ab52649.html
anti-Histone H3, Abcam, Cat#: ab1791
https://www.abcam.com/histone-h3-antibody-nuclear-marker-and-chip-grade-ab1791.html
anti-IGF-I (H-9), Santa Cruz, Cat#: sc-518040
https://www.scbt.com/p/igf-i-antibody-h-9
anti-IGF-I Receptor β, Cell Signaling, Cat#: 3027
https://www.cellsignal.com/products/primary-antibodies/igf-i-receptor-b-antibody/3027
anti-Lamin A/C (E-1), Santa Cruz, Cat#: sc-376248
https://www.scbt.com/p/lamin-a-c-antibody-e-1
anti-Lamin A + Lamin C, Abcam, Cat#: ab108595
https://www.abcam.com/lamin-a--lamin-c-antibody-epr4100-nuclear-envelope-marker-ab108595.html
anti-LAMP-2 (H4B4), Santa Cruz, Cat#: sc-18822
https://www.scbt.com/p/lamp-2-antibody-h4b4
anti-LAMP-2/CD107b, R&D, Cat#: AF6228
https://www.rndsystems.com/products/human-lamp-2-cd107b-antibody_af6228
anti-LAP1B, Novus, Cat#: NBP2-47403
https://www.novusbio.com/products/lap1b-antibody_nbp2-47403
anti-LC3B, Cell Signaling, Cat#: 2775
https://www.cellsignal.com/products/primary-antibodies/lc3b-antibody/2775
anti-c-Myc (9E10), Santa Cruz, Cat#: sc-40
https://www.scbt.com/p/c-myc-antibody-9e10
anti-Otx2 (EPR20375), Abcam, Cat#: ab183951
https://www.abcam.com/otx2-antibody-epr20375-ab183951.html
anti-Otx2, R&D, Cat#: AF1979
https://www.rndsystems.com/products/human-otx2-antibody_af1979
anti-p53 (FL-393), Santa Cruz, Cat#: sc-6243
https://www.scbt.com/p/p53-antibody-fl-393
anti-Parvalbumin, Novus, Cat#: NB120-11427
https://www.novusbio.com/products/parvalbumin-antibody_nb120-11427
anti-RFP, Abcam, Cat#: ab62341
https://www.abcam.com/rfp-antibody-ab62341.html
anti-SUN1, Novus, Cat#: NBP1-87396
https://www.novusbio.com/products/sun1-antibody_nbp1-87396
anti-Syndecan 3, Abcam, Cat#: ab36653
https://www.abcam.com/syndecan-3-antibody-ab36653.html
anti-Nesprin-2, Sigma-Aldrich, Cat#: ABT182
https://www.sigmaaldrich.com/KR/ko/product/mm/abt182
anti-Nesprin 2, Novus, Cat#: NBP1-84190
https://www.novusbio.com/products/nesprin-2-antibody_nbp1-84190
anti-Tom20 (FL-145), Santa Cruz, Cat#: sc-11415
https://www.scbt.com/p/tom20-antibody-fl-145
anti-Torsin A, Novus, Cat#: NBP2-95160
https://www.novusbio.com/products/torsin-a-antibody_nbp2-95160
anti-V (OASA04487), Aviva Systems Biology, Cat#: OASA04487
https://www.avivasysbio.com/v-antibody-oasa04487.html
anti-Wisteria floribunda agglutinin, Sigma-Aldrich, Cat#: L1516
https://www.sigmaaldrich.com/KR/ko/product/sigma/l1516

Eukaryotic cell lines

Policy information about cell lines and Sex and Gender in Research

Cell line source(s)	HeLa (catalog #: CCL-2) cell line was obtained from the American Type Culture Collection (ATCC).
Authentication	None
Mycoplasma contamination	Cell-lines used in this study was confirmed to be negative to mycoplasma contamination.
Commonly misidentified lines (See ICLAC register)	None

Animals and other research organisms

Policy information about studies involving animals; ARRIVE guidelines recommended for reporting animal research, and Sex and Gender in Research

Laboratory animals	Tor1aΔE/+ mice were purchased from the Jackson Laboratory (stock #: 025637).<br>LSL-KASH2 (Razafsky and Hodzic., 2014; https://doi.org/10.1002/dvg.22755) and FoxJ1-CreER (Rawlins et al., 2007; https://doi.org/10.1073/pnas.0610770104) mice were reported previously.<br>3-4 weeks old mice (C57BL/6 background) were used as described in this study.<br>All of the animals were maintained in a specific pathogen-free (SPF) facility of Korea Advanced Institute of Science and Technology (KAIST) under the 12-hour lights on/12-hour lights off cycle and at 22±2°C and 50±10% humidity.
Wild animals	None
Reporting on sex	Both male and female mice were used in this study.
Field-collected samples	None
Ethics oversight	All of the animals were handled according to approved institutional animal care and use committee (IACUC) protocols (#2012-37) of Korea Advanced Institute of Science and Technology (KAIST).

Note that full information on the approval of the study protocol must also be provided in the manuscript.
